# Hishot Display—A New Combinatorial Display for Obtaining Target-Recognizing Peptides

**DOI:** 10.1371/journal.pone.0083108

**Published:** 2013-12-27

**Authors:** Shoutaro Tsuji, Makiko Yamashita, Taihei Kageyama, Takashi Ohtsu, Katsuo Suzuki, Shintaro Kato, Joe Akitomi, Makio Furuichi, Iwao Waga

**Affiliations:** 1 Molecular Diagnostic Project, Kanagawa Cancer Center Research Institute, Yokohama, Japan; 2 Anticancer Drug Project, Kanagawa Cancer Center Research Institute, Yokohama, Japan; 3 VALWAY Technology Center, NEC Soft Ltd., Tokyo, Japan; Technical University of Braunschweig, Germany

## Abstract

Display technologies are procedures used for isolating target-recognizing peptides without using immunized animals. In this study, we describe a new display method, named Hishot display, that uses *Escherichia coli* and an expression plasmid to isolate target-recognizing peptides. This display method is based on the formation, in bacteria, of complexes between a polyhistidine (His)-tagged peptide including random sequences and the peptide-encoding mRNA including an RNA aptamer against the His-tag. When this system was tested using a sequence encoding His-tagged green fluorescent protein that included an RNA aptamer against the His-tag, the collection of mRNA encoding the protein was dependent on the RNA aptamer. Using this display method and a synthetic library of surrogate single-chain variable fragments consisting of VpreB and Ig heavy-chain variable domains, it was possible to isolate clones that could specifically recognize a particular target (intelectin-1 or tumor necrosis factor-α). These clones were obtained as soluble proteins produced by *E. coli*, and the purified peptide clones recognizing intelectin-1 could be used as detectors for sandwich enzyme-linked immunosorbent assays. The Hishot display will be a useful method to add to the repertoire of display technologies.

## Introduction

Target-recognizing molecules are widely used in research, disease diagnosis, and clinical treatment. Among such molecules, monoclonal antibodies (mAbs) are one of the most common for molecular recognition because of their specificity, affinity, and versatility. Most mAbs are isolated via hybridoma technology, which requires B cells in antigen-immunized animals [1]. However, immunization against toxic molecules, low-molecular-weight molecules, or highly conserved antigens is generally difficult. In addition, antibody production is unpredictable and time consuming. Furthermore, with traditional hybridoma technology, many immunized animals are killed for the extraction of lymphoid tissue, which raises concerns about animal ethics. To solve these problems, *in vitro* methods that isolate target-recognizing molecules, such as antibody fragments, without the need to use animals have been developed [2]. Phage display is an excellent example of such a procedure [3]. In phage display, a phage surface protein is fused with a variety of peptides with different target-binding regions, which can be made from randomly synthesized DNA or from a gene library (such as an antibody gene library) [4]. Many useful target-recognizing peptides, containing single-chain variable fragments (scFvs), were isolated via phage displays, especially those using filamentous bacteriophage [2]. Displays using bacteria [5], [6] or yeast [7] have also been developed, but are more restricted with regard to the variety of library and/or form of the target-recognizing peptide that can be used. Plasmid display, which is based on non-covalent binding between a DNA binding protein with short target-binding sequences and an expression plasmid encoding the protein of interest, has also been reported [8]–[10]. Ribosome display or mRNA display for isolating target-recognizing peptides has also been developed, which uses polysomes or the complexes that mRNA covalently binds to its translated peptides through puromycin, respectively [11]–[13]. Unlike the other methods, ribosome display and mRNA display use *in vitro* translation; thus, isolation of target-recognizing peptides is not hindered by target toxicity, size, or the presence of conserved sequences in the target. In addition, the peptides can be selected from high-variety libraries containing >10^10^ independent clones.

These established display methods have many advantages, but they may not be able to identify every target-recognizing peptide. Therefore, in order to resolve the difficulties and ethics concerns associated with animal immunization, additional display methods that do not require the use of animals should be developed. In this study, we describe a new display method, named Hishot display, that is based on basic molecular biological procedures using *Escherichia coli* and an expression plasmid. This display method uses the complex formed between a polyhistidine (His)-tagged peptide that contains random sequences and an mRNA that encodes the peptide with an RNA aptamer against the His-tag. With the Hishot display, the target-recognizing peptides were isolated as antibody fragments that could be obtained as soluble protein produced by *E. coli* and that bound the target specifically. The Hishot display is a useful method that should be added to the current repertoire of display technologies.

## Materials and Methods

### Plasmids

DNA fragments, including sequences of shot47, an RNA aptamer that binds to the His-tag [14], were inserted mainly between the *Nde*I and *Xba*I sites in a cold-shock expression vector, pCold IV (Takara Bio Inc., Shiga, Japan) [15]. The inserted fragments are shown in Figure S1. A His-tagged surrogate scFv consisting of human VpreB and human heavy-chain variable domain (V_H_) without complementarity-determining regions 3 (CDR3) was inserted into pCold IV with shot47; the resulting plasmid was named pHishot12. For the expression of the Fc-fused protein, we used an original mammalian expression vector, pHint, which has a human intelectin-1 signal peptide for secretion (Figure S2). A rabbit Fc-linked scFv with (VISF) or without (VIF) disulfide bridges was inserted into pHint (Figure S2). These plasmids were extracted by using the Qiagen Plasmid Midi Kit (Qiagen GmbH, Hilden, Germany), treated with Plasmid-Safe ATP-dependent DNase (Epicentre Biotechnologies, Madison, WI, USA), and purified by phenol–chloroform extraction and ethanol precipitation.

### Library for Hishot display

A plasmid library was made by inserting a synthetic CDR3 region containing random peptide sequences into pHishot12. Forward oligonucleotides, including 8 random codons (5′-GCTACGCTGCAGCGCGT VNKVNKVNKVNK TAT VNKVNKVNKVNK TTCGACTACTGGGGTCAGGGTAC-3′) (0.1 nmol), and reverse oligonucleotides (5′-CTAGTAGCGGCCGCTTATCTACCGCTGGAAACGGTAACCAGAGTACCCTGACCCCAGTAGTCGA-3′) (1 nmol) were mixed, and complementary strands were synthesized with Ex Taq (Takara Bio Inc.). The reaction conditions consisted of a denaturation step at 98°C for 30 s, followed by 5 cycles of 55°C for 1 min and 72°C for 1 min. The DNA was isolated by ethanol precipitation and treated with exonuclease I (Takara Bio Inc.) at 37°C for 18 h. Double-stranded DNA (dsDNA) was purified by phenol–chloroform extraction, digested with *Pst*I and *Not*I, resolved by agarose gel electrophoresis using 3% NuSieve GTG agarose (Takara Bio Inc.), extracted with thermostable β-agarase (Nippon Gene Co., Ltd., Tokyo, Japan), and purified by phenol extraction, phenol–chloroform extraction, and ethanol precipitation. The digested dsDNA (3 pmol) was inserted into *Pst*I- and *Not*I-digested pHishot12 (0.3 pmol) using T4 DNA ligase (Takara Bio Inc.) in a ligation buffer (60 mM Tris buffer (pH 7.5) containing 60 mM MgCl_2_, 50 mM NaCl, 70 mM 2-mercaptoethanol, 1 mM ATP, 20 mM dithiothreitol, 10 mM spermidine, and 1 mg/mL bovine serum albumin (BSA)) at 14°C for 18 h. The plasmid was purified by phenol–chloroform extraction, precipitated with 10 µg of tRNA by ethanol precipitation, and transferred into *E. coli* DH5α electrocompetent cells (Takara Bio Inc.) by electroporation (MicroPulser electroporator; Bio-Rad Laboratories, Inc., Hercules, CA, USA). The library (5–8×10^7^ colony-forming units (CFU)) was used for the Hishot display. Sequencing of 48 clones from a library indicated that the insert rate was >97% (47/48 clones) and that none of the clones contained a stop codon in their random region.

### Hishot display

*E. coli* transformed with the plasmid library was incubated in 100 mL of LB medium containing 100 µg/mL ampicillin at 37°C with continuous shaking to an OD_600_ of 0.6. After incubation at 10°C for 30 min, the bacteria was cultured with 0.5 mM isopropylthio-β-galactoside (IPTG) at 10°C with continuous shaking for 18 h. The following procedures were carried out at 4°C. The bacteria was centrifuged at 6,000× *g* for 10 min, washed with saline containing 10 mM ethylenediaminetetraacetic acid (EDTA), and incubated for 1 h with 20% sucrose (5 mL) containing 2 mg/mL lysozyme (Sigma-Aldrich Co., St. Louis, MO, USA), 1 mM EDTA, and 20 mM 4-(2-hydroxyethyl)-1-piperazineethanesulfonic acid (HEPES) (pH 7.5). The spheroid cells were washed gently with 20 mM HEPES-buffered saline (HBS) containing 0.1 mM magnesium acetate and then lysed with 4 mL of 20 mM HEPES (pH 7.5) containing 0.5% Triton-X100, 0.1 mM magnesium acetate, 100 µg/mL tRNA, 1 mg/mL BSA (A8577; Sigma-Aldrich Co.), 100 µg/mL rabbit IgG (I8140; Sigma-Aldrich Co.), 100 µg/mL human IgG (I8640; Sigma-Aldrich Co.), 100 µg/mL mouse IgG (I8765; Sigma-Aldrich Co.), 13 U/mL RNase inhibitor (Toyobo Co., Ltd., Osaka, Japan), 50 U/mL DNase I (Life Technologies, Carlsbad, CA, USA), and a protease inhibitor (Complete Mini EDTA-free; Roche Diagnostics GmbH, Mannheim, Germany). The lysate was passed twice through a syringe with a 27-gauge needle and then diluted in 40 mL of HBS containing 0.5% Triton-X100 and 0.1 mM magnesium acetate. The lysate was then centrifuged at 14,000× *g* for 10 min, after which the supernatant was mixed for 90 min with 20 µL of Affi-Gel 10 gel (Bio-Rad Laboratories, Inc.) coupled with human intelectin-1 or human tumor necrosis factor (TNF)-α. The gels were precipitated by centrifugation at 3,000× *g* for 5 min, washed twice with 40 mL of HBS containing 0.5% Triton-X100 and 0.1 mM magnesium acetate, and incubated for 5 min with 150 µL of TRIzol reagent (Life Technologies). The eluate was filtered through an Ultrafree-MC GV 0.22 µm filter (Merck Millipore, Billerica, MA, USA), and the RNA was purified according to the manufacturer's instructions and precipitated with 1 µL of Ethachinmate (Nippon Gene Co., Ltd.) in 50% isopropanol. The RNA was treated at 37°C for 30 min with RNase-free DNase (Promega Co., Madison, WI, USA) and purified by phenol–chloroform extraction and ethanol precipitation. The purified RNA clones were amplified by using a OneStep RT-PCR kit (Qiagen GmbH) in a 50 µL reaction mixture with 0.6 µM of primers (5′-CGGAAGACACGGCCGTTTATTACGCTG-3′ and 5′-CACTTAGCGGCCGCTTATCTACCGCTG-3′). The PCR conditions consisted of 20 cycles at 94°C for 30 s, 57°C for 30 s, and 72°C for 30 s. To obtain homologous dsDNA, excess primers (each 500 pmol) were added to the reaction mixture and incubated for 5 cycles at 55°C for 1 min and 72°C for 1 min following a prior denaturation at 98°C for 30 s. The dsDNA was collected by ethanol precipitation, treated with exonuclease I at 37°C for 18 h to digest excess primers, purified by phenol–chloroform extraction and ethanol precipitation, and solubilized in 10 µL of 10 mM Tris buffer (pH 7.0) containing 1 mM EDTA. The DNA (1 µL) was digested with *Pst*I and *Not*I at 37°C for 7 h, purified by phenol–chloroform extraction and ethanol precipitation, and solubilized in 3 µL of 10 mM Tris buffer (pH 7.0) containing 1 mM EDTA. The purified DNA (1.5 µL) was inserted into *Pst*I- and *Not*I-digested pHishot12 (0.3 pmol) using T4 DNA ligase in 50 µL of ligation buffer at 14°C for 18 h. The plasmid was purified by phenol–chloroform extraction, precipitated with 10 µg of tRNA by ethanol precipitation, and transferred into *E. coli* DH5α electrocompetent cells by electroporation (MicroPulser electroporator). The transformed *E. coli* was then used for the next round of Hishot display.

### Cloning and screening

After 2 or 3 rounds of Hishot display, the clones were isolated by picking up colonies from an LB-agar plate and cultured in 500 µL of LB medium containing 100 µg/mL ampicillin in a 96-deep-well plate with continuous shaking until the OD_600_ reading reached 0.4–0.6. After incubation at 10°C for 30 min, the bacteria were cultured with 0.5 mM IPTG at 10°C for 18 h by continuous shaking. The cultures were centrifuged at 2,300× *g* for 20 min, and the supernatants were carefully removed by pipetting. The cells were lysed by freeze-thawing in 200 µL of 10 mM HEPES (pH 7.5) containing 0.5% Triton-X100, 1 mM EDTA, 13 U/mL benzonase nuclease (Merck KGaA, Darmstadt, Germany), and 500 U/mL rLysozyme (Merck KGaA), and then 5 µL of 100 mM magnesium acetate was added. The mixture was then centrifuged at 2,300× *g* for 5 min, and the supernatants were screened by enzyme-linked immunosorbent assay (ELISA) as follows. Supernatants were diluted 3 times with 225 mM NaCl and incubated at 25°C for 2 h on an ELISA plate that had been precoated with 5 µg/mL of a target protein and blocked with 1% BSA. The plate was washed 3 times with Tris-buffered saline containing 0.1% Tween-20 and incubated at 25°C for 1 h with a horseradish peroxidase-conjugated anti-His-tag mAb (Penta·His HRP Conjugate; Qiagen GmbH) diluted 1∶3,000. The plate was washed 3 times with Tris-buffered saline containing 0.1% Tween-20 and incubated for 9 min with 1-Step Ultra TMB (Thermo Fisher Scientific Inc., Rockford, IL, USA). The reaction was terminated by adding H_2_SO_4_, and the absorbance was then measured at 450 nm. Clones that bound nonspecifically to rabbit IgG were excluded.

### Purification of surrogate scFv

*E. coli* DH5α cells transformed with the selected plasmid were cultured to an OD_600_ of 0.6 in 100 mL of LB medium containing 100 µg/mL ampicillin. Production of His-tagged surrogate scFv was then induced with 0.5 mM IPTG at 10°C for 24 h. The cells were collected by centrifugation at 6,000× *g* for 10 min, and lysed by freeze-thawing with 4 mL of 10 mM HEPES (pH 8.0) containing 0.5% Triton-X100, 1 mM EDTA, 13 U/mL benzonase nuclease, 500 U/mL rLysozyme, and a protease inhibitor (Complete Mini EDTA-free), and then 1 M magnesium acetate (20 µL) was added. After the lysate was incubated at 4°C for 30 min, the supernatant was collected by centrifugation at 14,000× *g* for 10 min and diluted with an equal volume of 10 mM HEPES (pH 8.0) containing 300 mM NaCl and 5 mM imidazole. The sample was applied to a Ni-NTA agarose column (1 mL; Qiagen GmbH) equilibrated with HBS (pH 8.0) containing 20 mM imidazole. The column was washed with HBS (pH 8.0) containing 20 mM imidazole, and the His-tagged surrogate scFv was eluted with HBS (pH 8.0) containing 250 mM imidazole. The surrogate scFv was dialyzed against HBS, and its concentration was calculated from its coefficient of absorbance at 280 nm [16].

### Rabbit Fc-fused protein

The plasmid of the isolated clone was used as the template DNA. The random region of the clone was amplified by PCR using the following primers: 5′-CGGAAGACACGGCCGTTTATTACGCTG-3′ and 5′-CTAGTAGCGGCCGCTGGAAACGGTAACCAGAGT-3′ (VIF) or 5′-AGGACACTGCAGTTTATTACTGTGCAGCGCGT-3′ and 5′-CTAGTAGCGGCCGCTGGAAACGGTAACCAGAGT-3′ (VISF). The PCR products were digested with *Pst*I and *Not*I and inserted into VIF/pHint or VISF/pHint, respectively (Figure S2). The purified plasmid was transfected into the rabbit kidney RK-13 cell line (obtained from RIKEN cell bank (Tsukuba, Japan)) with Lipofectamine 2000 (Life Technologies), and the culture supernatant was used as the sample containing the Fc-fused protein. Secretion of the Fc-fused protein was confirmed by western blot analysis using anti-rabbit IgG (data not shown).

## Results

### System design

**Figure 1:**
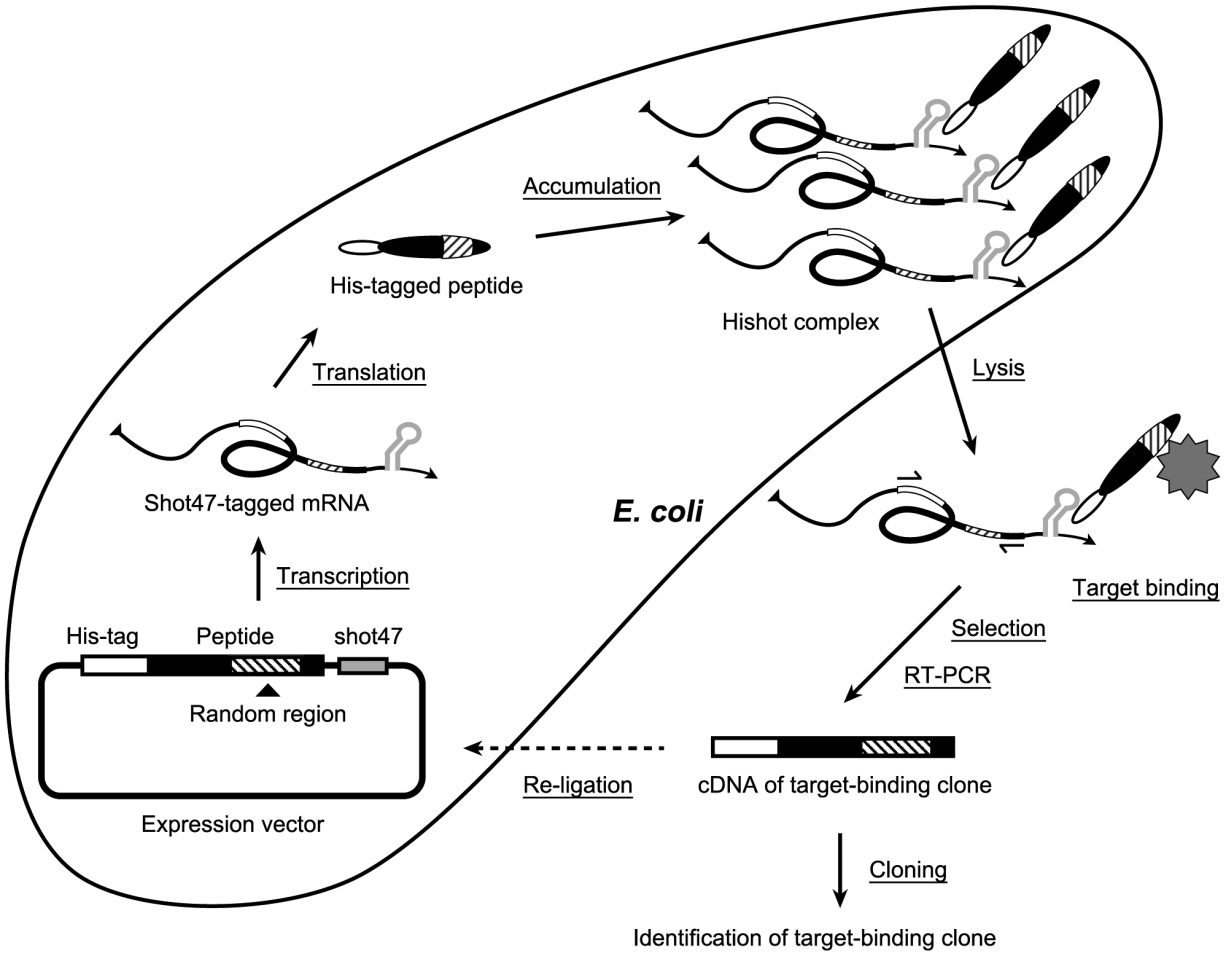
Schema of Hishot display. Shot47-tagged mRNA is transcribed from an expression vector, in which a gene encoding the His-tagged peptide, including shot47 sequences, has been inserted. The Hishot complex is formed by combination of the translated His-tagged peptide and the shot47-tagged mRNA, and multiple Hishot complexes accumulate in a single bacterium. After the Hishot complex has been harvested via bacterial lysis and selection, the mRNA is amplified with RT-PCR and its sequence is identified by cloning.

The RNA aptamer, shot47, binds strongly to the His-tag with a low picomolar dissociation constant [14]. Furthermore, shot47 can form a stable complex with His-tagged protein in bacterial lysates. Therefore, we hypothesized that shot47-tagged mRNA encoding His-tagged peptides would stably associate with the translated His-tagged peptides in bacteria. Since only a single plasmid clone is amplified per bacterium, after transformation of bacteria with a tagged plasmid library, the shot47-tagged mRNA transcribed from the plasmid in each bacterium would interact only with its translated His-tagged peptide (Figure 1). In this way, multiple complexes of **Hi**s-tagged peptides and **shot**47-tagged mRNA (Hishot complexes) can be formed in bacteria and then solubilized by bacterial lysis. After a target-binding Hishot complex has been isolated, its mRNA sequence can be amplified by RT-PCR and then cloned and sequenced to identify the target-binding peptide. We named this method the Hishot display as it uses Hishot complex.

### Evaluation of system

**Figure 2:**
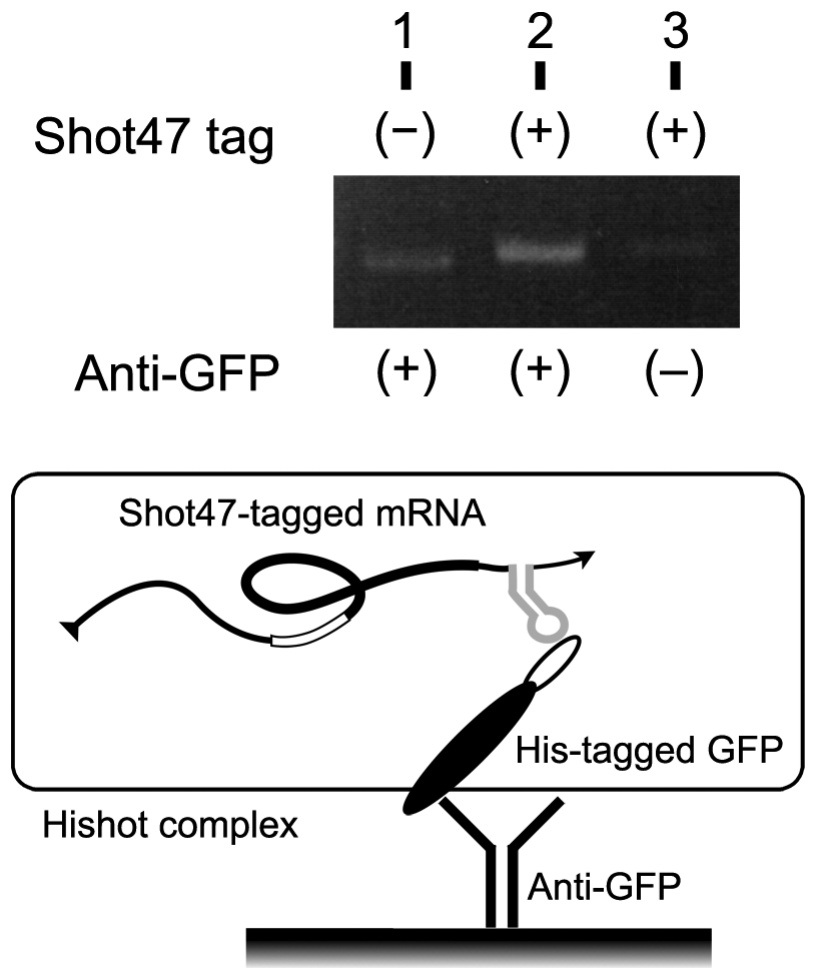
Collection of mRNA via the shot47 tag. A gene encoding His-tagged GFP including shot47 sequences was inserted into the pHishot1 expression vector. GFP expression was induced, and the Hishot complex was then precipitated with anti-GFP and amplified by RT-PCR. Lane 1, collection of mRNA without shot47 sequences; lane 2, collection of shot47-tagged mRNA; lane 3, nonspecific collection of shot47-tagged mRNA without anti-GFP.

**Figure 3:**
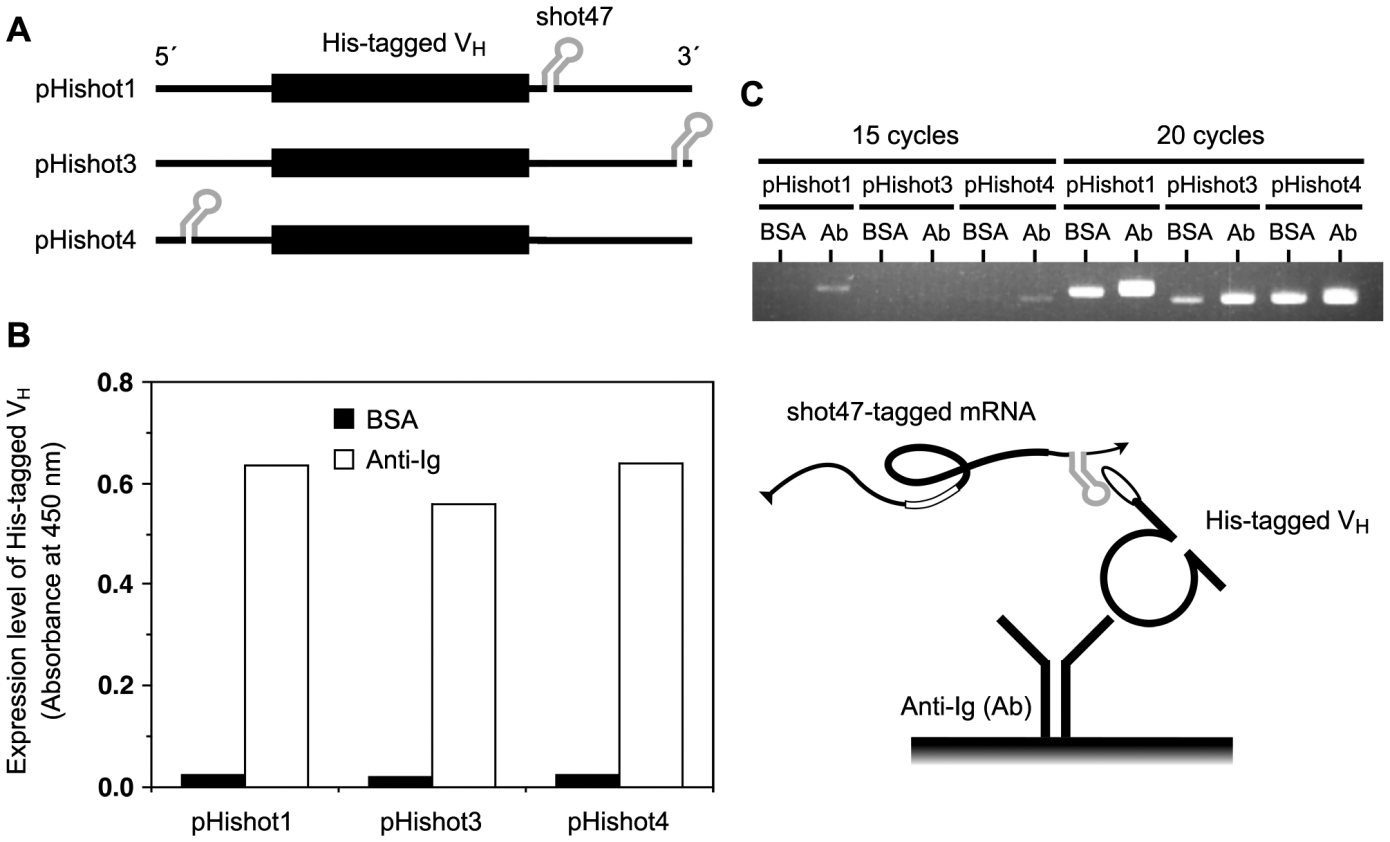
Determining the best position for shot47 for collecting the Hishot complex. (A) Schema of the possible positions for shot47. Shot47 sequences were inserted into a gene of the His-tagged V_H_ fragment at 3 different positions (Figure S1). (B) Expression of His-tagged V_H_ from expression vector containing shot47. The expression level was measured by sandwich ELISA using anti-Ig antibody (capture) and horseradish peroxidase-conjugated anti-His-tag mAb (detector). (C) Amount of mRNA in the Hishot complex on a plate coated with BSA or anti-Ig (Ab). The collected mRNA was amplified by RT-PCR (15 or 20 cycles of amplification), resolved by agarose gel electrophoresis, and stained with ethidium bromide.

**Figure 4:**
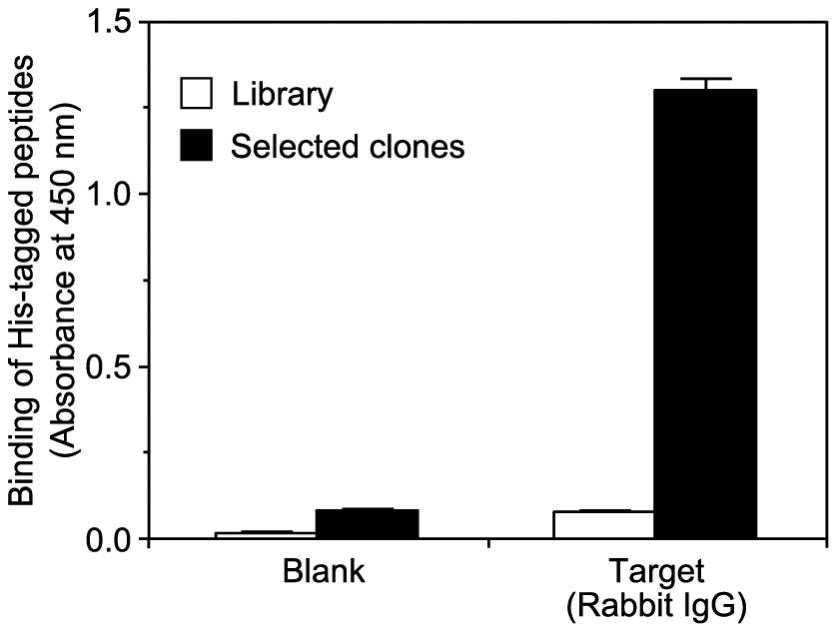
Concentration of target-binding clones by Hishot display using a random peptide. Rabbit IgG was used as a target. Hishot complexes in a library were collected with rabbit IgG on the plate, amplified by RT-PCR, and then re-ligated into pHishot1. Clones in the next round were shown as selected clones. The amount of binding peptide was measured by ELISA using rabbit IgG (target) and horseradish peroxidase-conjugated anti-His-tag mAb (detector). Values represent the mean ± S.D. of triplicate determinations.

To examine the shot47 dependence of a collection of Hishot complexes, a construct containing a His-tagged green fluorescent protein (GFP) with shot47 sequences in its 3′ untranslated region (UTR) was used (Figure 2). By affinity adsorption with anti-GFP antibody, more shot47-tagged mRNAs (lane 2) were collected than mRNAs without shot47 (lane 1) or mRNAs of Hishot complexes that were nonspecifically adsorbed on the matrix (lane 3). This result suggested that the Hishot complex collected was shot47-dependent. To determine the most effective position of shot47 for collecting the Hishot complex, the shot47 sequence was inserted into the gene of the His-tagged V_H_ fragment at 3 different positions: pHishot1, in the 3′ UTR just after the stop codon and upstream of the terminator sequence; pHishot3, in the terminator sequence site in 3′ UTR; and pHishot4, in the 5′ UTR just after the transcription initiation site (Figure 3A and Figure S1). Although the position of the shot47 sequence did not affect translation of the peptide (Figure 3B), the highest amount of Hishot complex was obtained with anti-Ig when pHishot1 was used (Figure 3C). Therefore, we used pHishot1 as the basis for constructing the Hishot display using the V_H_ fragment. To test the Hishot display system, we tried to isolate the target-binding clones from a library of His-tagged peptides consisting of 20 random residues (Figure 4 and Figure S1). Although target-binding clones were enriched in the peptide mixture selected once by Hishot display (Figure 4), we were unable to isolate an individual target-binding clone from this peptide pool (see Discussion).

### Isolation of target-binding clone from library

**Figure 5:**
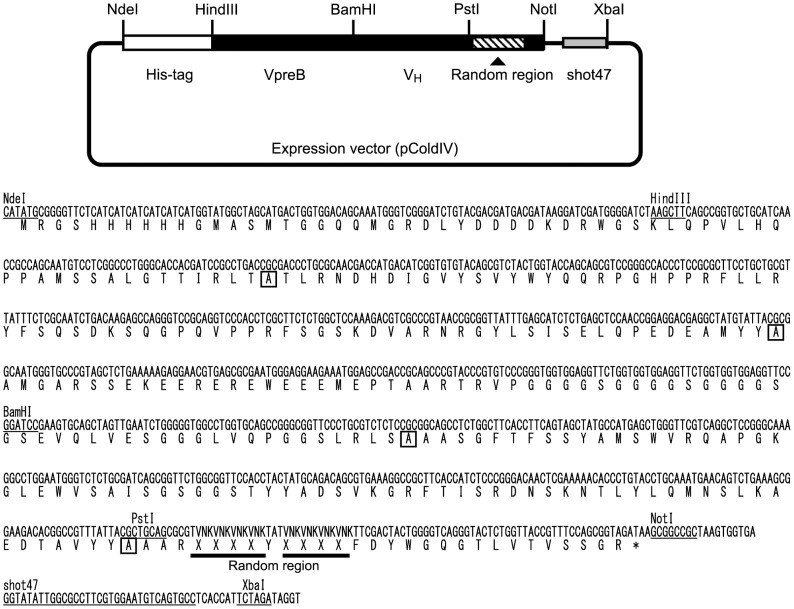
Structure of the expression vector for Hishot display. The schema of library plasmid and sequence of the insert, including the surrogate scFv and shot47, are shown. This plasmid was named pHishot12. Ala substituted for Cys is indicated by a square box. The main restriction enzyme or shot47 sequences are underlined. The random sequence region is shown by the bold underline.

To ensure the isolation of the clone, we tried using some antibody fragments, such as V_H_, the llama VHH fragment [17], or scFv, including short random sequences. We eventually used a surrogate scFv; namely, human VpreB (which can be used in place of the light-chain variable domain [18], [19]) that was linked with human V_H_ through a peptide linker, (GGGGS)_3_. The surrogate scFv was more suitable than the other tested Ig fragments for obtaining a clone with good specificity and binding activity (data not shown). Figure 5 shows the structure of the expression vector (pHishot12) used for the Hishot display plasmid library. A synthetic gene encoding the His-tagged surrogate scFv, including shot47 after the stop codon, was inserted into an *E. coli* expression vector, pCold IV (see Materials and Methods). To avoid the formation of an irregular disulfide-linked polymer, Cys residues in this surrogate scFv were replaced with Ala, as antibodies with Ala substituted for Cys often retain their target-binding activity [20]. The plasmid library was made by inserting various synthetic CDR3 regions including a variety of random peptide sequences into pHishot12. The random peptide region does not contain Cys, Phe, or Trp, and is free from stop codons as it only uses codons of the form VNK, where V is C, G, or A, N is any base, and K is T or G (see Materials and Methods).

**Figure 6:**
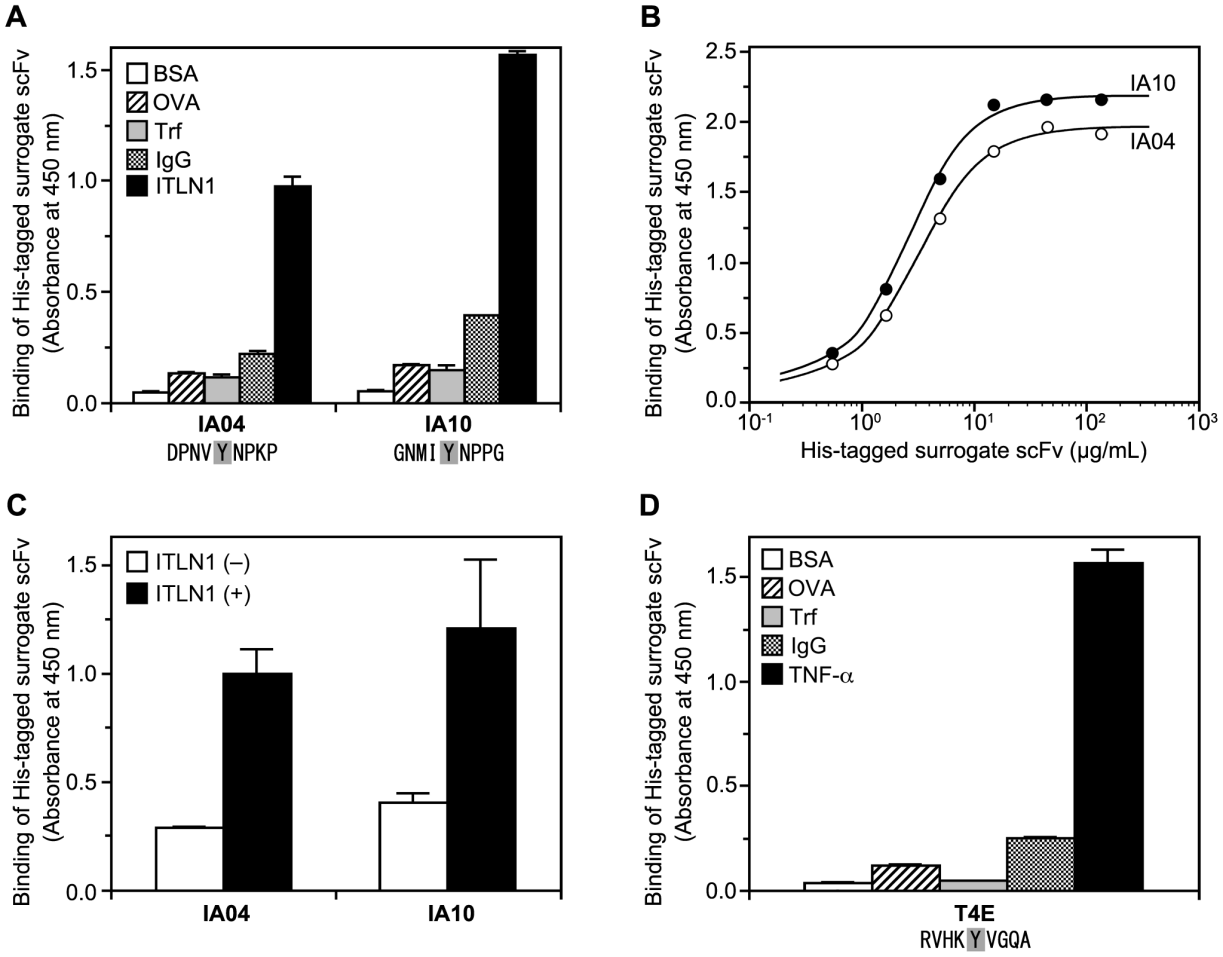
Target-binding activity of surrogate scFv isolated by Hishot display. Binding of surrogate scFv was measured by ELISA using horseradish peroxidase-conjugated anti-His-tag mAb. The ELISA plate was coated with proteins (5 µg/mL) and then blocked with BSA. Values represent the mean ± S.D. of triplicate determinations. (A) Specific binding activity of surrogate scFv against intelectin-1. The amino acid sequence of the random region is shown under the clone name (IA04 and IA10). Fixed Tyr is indicated as shaded characters. (B) Dose-dependency of the binding of purified surrogate scFv against intelectin-1. Values represent the mean of duplicate determinations. (C) Sandwich ELISA against intelectin-1, using surrogate scFv against intelectin-1 as the detector. Anti-intelectin-1 (capture mAb, 10:2D2) was coated at a concentration of 2 µg/mL, and intelectin-1 (0.1 µg/mL) was captured. The surrogate scFv against intelectin-1 was used at the concentration of 8 µg/mL. (D) Specific binding activity of surrogate scFv against TNF-α. The amino acid sequence of the random region is shown under the clone name (T4E). Fixed Tyr is indicated as shaded characters. BSA, bovine serum albumin; OVA, ovalbumin; Trf, transferrin; IgG, rabbit IgG; ITLN1, human intelectin-1; TNF-α, human tumor necrosis factor-α.

Figure 6A shows the specific binding activity against intelectin-1 of surrogate scFv clones isolated by Hishot display using the pHishot12 library. Two clones, IA04 and IA10, bound to intelectin-1 significantly more than to BSA, ovalbumin, transferrin, or rabbit IgG, and these clones had similar amino acid sequences in their random regions. Purified clones also bound to intelectin-1 adsorbed on a plate, and the bindings saturated at about 10 µg/mL (Figure 6B). In addition, it was possible to use these surrogate scFv clones as detectors for intelectin-1 in sandwich ELISAs (Figure 6C). Figure 6D shows that a clone isolated by Hishot display against TNF-α could bind to TNF-α specifically; in this clone, the sequences in the random region were quite different from those in the intelectin-1-binding clones. These results indicated that, using the Hishot display, it is possible to obtain surrogate scFv clones that specifically bind to a chosen target.

**Figure 7:**
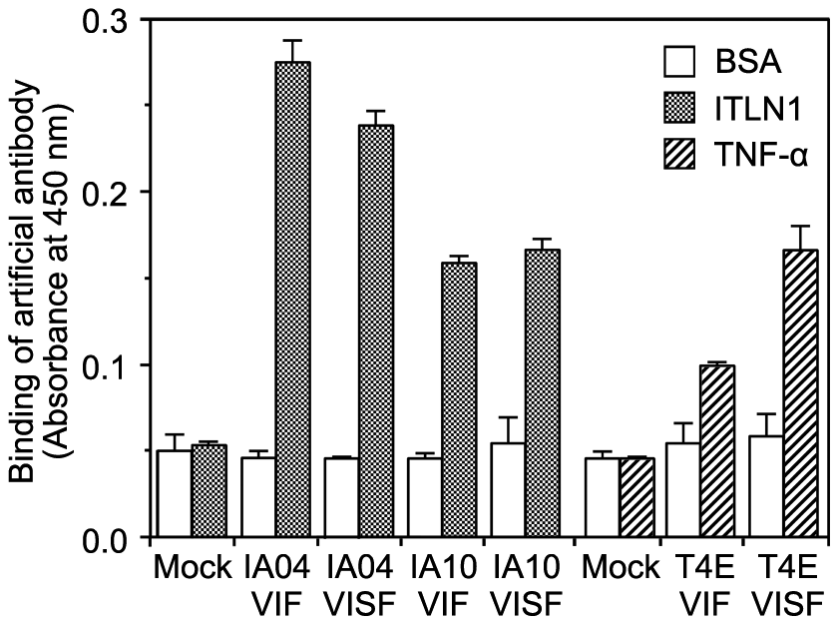
Target-binding activity of Fc-linked surrogate scFv against intelectin-1 or TNF-α. Binding of Fc-linked surrogate scFv was measured by ELISA using horseradish peroxidase-conjugated anti-rabbit IgG. The ELISA plate was coated with proteins (5 µg/mL) and then blocked with BSA. The supernatant from a culture of plasmid-transfected cells was used as the sample. The culture supernatant of secretory GFP-transfected cells was used as the mock control. The sequences of VIF or VISF are described in Figure S2. Values represent the mean ± S.D. of triplicate determinations. BSA, bovine serum albumin; ITLN1, human intelectin-1; TNF-α, human tumor necrosis factor-α.

The isolated surrogate scFv clones could be largely and easily purified as soluble proteins from transformed *E. coli*. However, after a long term storage (>1 month) at 4°C, they aggregated and precipitated from solution. To test the effect of fusion with the Fc region on storage stability, the clones were fused with the rabbit Fc region, and the Cys residues in VpreB and V_H_ were reconstituted for formation of the disulfide bridge (Figure S2, VISF/pHint). The recombinant Fc-linked surrogate scFv was expressed in rabbit kidney cell lines, which secreted it into the culture supernatant. As shown in Figure 7, Fc-linked surrogate scFvs against intelectin-1 or TNF-α were able to bind to their respective targets. The binding activities of the Fc-linked surrogate scFvs against intelectin-1 were not significantly affected by the formation of the disulfide bridge in the scFv region (Figure 7, VIF or VISF), whereas the disulfide bridge in the scFv region of Fc-linked surrogate scFv against TNF-α increased binding to the target (Figure 7, T4E-VISF). Regrettably, the recombinant Fc-linked proteins used in this study could not be purified as stable protein that retained activity after long-term storage (data not shown). However, these results indicated that the surrogate scFvs isolated by the Hishot display can re-form into soluble dimeric antibodies, and that disulfide bridge may improve the activity of these surrogate scFvs.

## Discussion

Hishot display has the following advantages, as follows: (1) it relies on basic molecular biological procedures using *E. coli* and an expression plasmids; (2) most soluble peptides that can be produced by *E. coli* can be used as the base for target-recognizing peptides in a Hishot display, and the isolated peptide can, in principle, be largely purified; and (3) the method for selection of the target-recognizing peptide is not limited to panning, because the Hishot complex is small and stable; furthermore, it is possible to isolate clones of target-recognizing peptides efficiently, because many Hishot complexes can be formed and accumulated in bacteria.

On the other hand, one disadvantage is that there are fewer libraries available for Hishot display than that for phage or ribosome displays. However, a library with the titer of 10^8^ CFU, which is comparable to that of B cells in mice [21], can be easily made by electroporation. Given that mice can produce many monoclonal antibodies against a target, this lesser but comparable variety would not be at an acute disadvantage. Another possible disadvantage would be template switching potentially resulting from non-covalent binding between the His-tagged peptide and the shot47-tagged mRNA. This could be a serious problem if only a small number of complexes of each clone were formed and the dissociation constant for the binding affinity between the peptide and mRNA was not small enough, which may affect the efficiency of isolating target-recognizing peptides. However, in Hishot display, many different complexes can be formed in exponentially propagating bacteria. In addition, the RNA aptamer used in this study, shot47, has such a small dissociation constant (<3.78 pM) that it is difficult to measure; in fact, the dissociation between the aptamer and the His-tag cannot be detected [14]. Moreover, in this study, we showed that the clones recognizing individual targets were isolated by using Hishot display. Thus, the template switching would not be a major issue interfering with Hishot display.

Hishot display also has some additional problems that are still pending, which are difficulties common to all display methods. The reactivity of the isolated clone was strongly affected by the method of selection and screening. In particular, screening method affected the binding ability of clone, in that a clone that had a higher affinity in the target-coated ELISA did not bind specifically in the sandwich ELISA or with other methods for detection of target-binding (data not shown). Furthermore, the isolation efficiency for the target-binding clone was also influenced by various factors, such as the salt concentration of the buffer, the washing condition, and the procedure of selection (e.g., panning, pull-down with agarose beads, or pull-down with polystyrene beads). These problems might be caused by the inflexibility and instability of the base peptide containing random regions, rather than by any problems with the Hishot display. Although the surrogate scFv used in this study had relatively good specificity and binding activity, it was not able to isolate a strong binder that could work in multiple detection methods. On display methods that don't use animals, affinity maturation is often required to obtain a strong binder. The use of a carrier protein containing multiple random regions, such as a normal scFv containing both V_H_ and V_L_ or a non-immunoglobulin protein [22], may be effective in obtaining more stable and versatile target-recognizing peptides.

Although target-binding peptides could be concentrated via the Hishot display by using random peptides (Figure 4), individual clones could not be isolated from the peptide pool. Moreover, the results obtained using the Hishot display with random peptides were not reproducible; this may be because the number of possible combinations from a random mixture of 20 amino acids (>10^26^) is too large to screen in full, with the total weight of all peptides that could potentially be produced amounting to >300 kg. Since the number of clones that can be screened by Hishot or other display methods is <1/10^13^ only, it may be difficult to isolate specific clones reproducibly. Furthermore, many of the peptides in the selected pool that were not screened contained hydrophobic amino acids (data not shown). Although these homogeneous peptides would have been at a low concentrations overall in a lysate made from a pool of multiple clones, lysate from a single peptide clone would contain a high concentration of such peptides. In such cases, the peptides might aggregate with one another and become insoluble, therefore going undetected in the screening. To avoid possible aggregation and insolubilization of candidate peptides in the Hishot display, it would be necessary to surround the random peptide sequence with a hydrophilic carrier protein, such as a variable domain of the antibody. Proper selection of the hydrophilic carrier peptide may be important to ensure isolation of the target-recognizing peptides by Hishot display.

## Supporting Information

Figure S1 **Sequences of genes inserted into the bacterial expression vector.** The inserts are indicated as sequences between the Lac operator and terminator in pCold IV. The main restriction enzyme or shot47 sequences are underlined. The X in the amino acid sequences indicates an undetermined amino acid. V, N, or K in the DNA sequences indicates mixed bases as follows: V, C/G/A; N, T/C/A/G; K, T/G.
(PDF)

Figure S2 **Sequences and structure of mammalian expression vectors.** The structure of the pHint expression vector is shown as a schema. Sequences of the antibody gene in VIF/pHint and VISF/pHint are shown from the signal peptide of human intelectin-1 (hITLN1) to the stop codon. The main restriction enzyme or signal peptide sequences are underlined. The X in the amino acid sequences indicates an undetermined amino acid. V, N, or K of the DNA sequences indicates mixed bases as follows: V, C/G/A; N, T/C/A/G; K, T/G.
(PDF)
