# Supplementary material for: Hishot Display—A New Combinatorial Display for Obtaining Target-Recognizing Peptides
Source: PLoS One. 2013 Dec 27;8(12):e83108. doi: 10.1371/journal.pone.0083108 (PMC3873924; doi:10.1371/journal.pone.0083108)
Supplement: Figure S1 — Sequences of genes inserted into the bacterial expression vector. The inserts are indicated as sequences between the Lac operator and terminator in pCold IV. The main restriction enzyme or shot47 sequences are underlined. The X in the amino acid sequences indicates an undetermined amino acid. V, N, or K in the DNA sequences indicates mixed bases as follows: V, C/G/A; N, T/C/A/G; K, T/G. (PDF) [file pone.0083108.s001.pdf]

His-tagged GFP

```
Lac operator               NheI
AATTGTGAGCGGATAACAATTTGATGTGCTAGCGCATATCCAGTGTAGTAAGGCAAGTCCCTTCAAGAGTTATCGTTGATACCCCTCGTAGTGCACATTCCTTTAACGCTT

                                                            NdeI
CAAAATCTGTAAAGCACGCCATATCGCCGAAAGGCACACTTAATTATTAAGAGGTAATACCATATGCGGGGTTCTCATCATCATCATCATCATGGTATGGCTAGCATGACT
                                                               M  R  G  S  H  H  H  H  H  H  G  M  A  S  M  T

GGTGGACAGCAAATGGGATCCATCGCCACCATGGTGAGCAAGGGCGAGGAGCTGTTCACCGGGGTGGTGCCCATCCTGGTCGAGCTGGACGGCGACGTAAACGGCCACAAG
G  G  Q  Q  M  G  S  I  A  T  M  V  S  K  G  E  E  L  F  T  G  V  V  P  I  L  V  E  L  D  G  D  V  N  G  H  K

TTCAGCGTGTCCGGCGAGGGCGAGGGCGATGCCACCTACGGCAAGCTGACCCTGAAGTTCATCTGCACCACCGGCAAGCTGCCCGTGCCCTGGCCCACCCTCGTGACCACC
F  S  V  S  G  E  G  E  G  D  A  T  Y  G  K  L  T  L  K  F  I  C  T  T  G  K  L  P  V  P  W  P  T  L  V  T  T

CTGACCTACGGCGTGCAGTGCTTCAGCCGCTACCCCGACCACATGAAGCAGCACGACTTCTTCAAGTCCGCCATGCCCGAAGGCTACGTCCAGGAGCGCACCATCTTCTTC
L  T  Y  G  V  Q  C  F  S  R  Y  P  D  H  M  K  Q  H  D  F  F  K  S  A  M  P  E  G  Y  V  Q  E  R  T  I  F  F

AAGGACGACGGCAACTACAAGACCCGCGCCGAGGTGAAGTTCGAGGGCGACACCCTGGTGAACCGCATCGAGCTGAAGGGCATCGACTTCAAGGAGGACGGCAACATCCTG
K  D  D  G  N  Y  K  T  R  A  E  V  K  F  E  G  D  T  L  V  N  R  I  E  L  K  G  I  D  F  K  E  D  G  N  I  L

GGGCACAAGCTGGAGTACAACTACAACAGCCACAACGTCTATATCATGGCCGACAAGCAGAAGAACGGCATCAAGGTGAACTTCAAGATCCGCCACAACATCGAGGACGGC
G  H  K  L  E  Y  N  Y  N  S  H  N  V  Y  I  M  A  D  K  Q  K  N  G  I  K  V  N  F  K  I  R  H  N  I  E  D  G

AGCGTGCAGCTCGCCGACCACTACCAGCAGAACACCCCCATCGGCGACGGCCCCGTGCTGCTGCCCGACAACCACTACCTGAGCACCCAGTCCGCCCTGAGCAAAGACCCC
S  V  Q  L  A  D  H  Y  Q  Q  N  T  P  I  G  D  G  P  V  L  L  P  D  N  H  Y  L  S  T  Q  S  A  L  S  K  D  P

                                                                                     NotI              shot47
AACGAGAAGCGCGATCACATGGTCCTGCTGGAGTTCGTGACCGCCGCCGGGATCACTCTCGGCATGGACGAGCTGTACAAGTAAAGCGGCCGCTAAGTGGTGAGGTATATT
N  E  K  R  D  H  M  V  L  L  E  F  V  T  A  A  G  I  T  L  G  M  D  E  L  Y  K  *

                                XbaI                                     Terminator
GGCGCCTTCGTGGAATGTCAGTGCCTCACCATTCTAGATAGGTAATCTCTGCTTAAAAGCACAGAATCTAAGATCCCTGCCATTTGGCGGGGATTTTTTTATTTGTTTTCA
```

pHishot1

```
Lac operator               NheI
AATTGTGAGCGGATAACAATTTGATGTGCTAGCGCATATCCAGTGTAGTAAGGCAAGTCCCTTCAAGAGTTATCGTTGATACCCCTCGTAGTGCACATTCCTTTAACGCTT

                                                            NdeI
CAAAATCTGTAAAGCACGCCATATCGCCGAAAGGCACACTTAATTATTAAGAGGTAATACCATATGCGGGGTTCTCATCATCATCATCATCATGGTATGGCTAGCATGACT
                                                               M  R  G  S  H  H  H  H  H  H  G  M  A  S  M  T

GGTGGACAGCAAATGGGTCGGGATCTGTACGACGATGACGATAAGGATCGATGGGGATCCCAGGTGCAGCTACAAGAATCTGGGGGTGGCCTGGTGCAGGCGGGCGGTTCC
G  G  Q  Q  M  G  R  D  L  Y  D  D  D  D  K  D  R  W  G  S  Q  V  Q  L  Q  E  S  G  G  G  L  V  Q  A  G  G  S

CTGCGTCTCTCCGCGGCAGCCTCTGGCCGCACCTTCAGTAGCTATGGCATGGGCTGGTTTCGTCAGGCTCCGGGCAAAGAACGTGAATTCGTCGCAGCGATCAGCTGGTCT
L  R  L  S  A  A  A  S  G  R  T  F  S  S  Y  G  M  G  W  F  R  Q  A  P  G  K  E  R  E  F  V  A  A  I  S  W  S

GGCGGTTCCACCTACTATGCAGACAGCGTGAAAGGCCGCTTCACCATCTCCCGGGACAACGCGAAAAACACCGTGTACCTGCAAATGAACAGTCTGAAACCGGAAGACACG
G  G  S  T  Y  Y  A  D  S  V  K  G  R  F  T  I  S  R  D  N  A  K  N  T  V  Y  L  Q  M  N  S  L  K  P  E  D  T

             PstI           NotI              shot47                                  XbaI
GCCGTTTATTACGCTGCAGCGGTTTCCAGCGGCCGCTAAGTGGTGAGGTATATTGGCGCCTTCGTGGAATGTCAGTGCCTCACCATTCTAGATAGGTAATCTCTGCTTAAA
A  V  Y  Y  A  A  A  V  S  S  G  R  *

                Terminator
AGCACAGAATCTAAGATCCCTGCCATTTGGCGGGGATTTTTTTATTTGTTTTCA
```

pHishot3

```
Lac operator               NheI
AATTGTGAGCGGATAACAATTTGATGTGCTAGCGCATATCCAGTGTAGTAAGGCAAGTCCCTTCAAGAGTTATCGTTGATACCCCTCGTAGTGCACATTCCTTTAACGCTT

                                                            NdeI
CAAAATCTGTAAAGCACGCCATATCGCCGAAAGGCACACTTAATTATTAAGAGGTAATACCATATGCGGGGTTCTCATCATCATCATCATCATGGTATGGCTAGCATGACT
                                                               M  R  G  S  H  H  H  H  H  H  G  M  A  S  M  T

GGTGGACAGCAAATGGGTCGGGATCTGTACGACGATGACGATAAGGATCGATGGGGATCCCAGGTGCAGCTACAAGAATCTGGGGGTGGCCTGGTGCAGGCGGGCGGTTCC
G  G  Q  Q  M  G  R  D  L  Y  D  D  D  D  K  D  R  W  G  S  Q  V  Q  L  Q  E  S  G  G  G  L  V  Q  A  G  G  S

CTGCGTCTCTCCGCGGCAGCCTCTGGCCGCACCTTCAGTAGCTATGGCATGGGCTGGTTTCGTCAGGCTCCGGGCAAAGAACGTGAATTCGTCGCAGCGATCAGCTGGTCT
L  R  L  S  A  A  A  S  G  R  T  F  S  S  Y  G  M  G  W  F  R  Q  A  P  G  K  E  R  E  F  V  A  A  I  S  W  S

GGCGGTTCCACCTACTATGCAGACAGCGTGAAAGGCCGCTTCACCATCTCCCGGGACAACGCGAAAAACACCGTGTACCTGCAAATGAACAGTCTGAAACCGGAAGACACG
G  G  S  T  Y  Y  A  D  S  V  K  G  R  F  T  I  S  R  D  N  A  K  N  T  V  Y  L  Q  M  N  S  L  K  P  E  D  T

             PstI           NotI       XbaI                                     Terminator   shot47
GCCGTTTATTACGCTGCAGCGGTTTCCAGCGGCCGCTAATCTAGATAGGTAATCTCTGCTTAAAAGCACAGAATCTAAGATCCCTGCCAGGTATATTGGCGCCTTCGTGGA
A  V  Y  Y  A  A  A  V  S  S  G  R  *

ATGTCAGTGCCTGGCGGGGATTTTTTTATTTGTTTTCA
```

pHishot4

```
Lac operator                          shot47                                NheI
AATTGTGAGCGGATAACAATTTGATGTGCTAGTGGTGAGGTATATTGGCGCCTTCGTGGAATGTCAGTGCCTCACCGCTAGCGCATATCCAGTGTAGTAAGGCAAGTCCCT

TCAAGAGTTATCGTTGATACCCCTCGTAGTGCACATTCCTTTAACGCTTCAAAATCTGTAAAGCACGCCATATCGCCGAAAGGCACACTTAATTATTAAGAGGTAATAC

NdeI
CATATGCGGGGTTCTCATCATCATCATCATCATGGTATGGCTAGCATGACTGGTGGACAGCAAATGGGTCGGGATCTGTACGACGATGACGATAAGGATCGATGGGGATCC
   M  R  G  S  H  H  H  H  H  H  G  M  A  S  M  T  G  G  Q  Q  M  G  R  D  L  Y  D  D  D  D  K  D  R  W  G  S

CAGGTGCAGCTACAAGAATCTGGGGGTGGCCTGGTGCAGGCGGGCGGTTCCCTGCGTCTCTCCGCGGCAGCCTCTGGCCGCACCTTCAGTAGCTATGGCATGGGCTGGTTT
Q  V  Q  L  Q  E  S  G  G  G  L  V  Q  A  G  G  S  L  R  L  S  A  A  A  S  G  R  T  F  S  S  Y  G  M  G  W  F

CGTCAGGCTCCGGGCAAAGAACGTGAATTCGTCGCAGCGATCAGCTGGTCTGGCGGTTCCACCTACTATGCAGACAGCGTGAAAGGCCGCTTCACCATCTCCCGGGACAAC
R  Q  A  P  G  K  E  R  E  F  V  A  A  I  S  W  S  G  G  S  T  Y  Y  A  D  S  V  K  G  R  F  T  I  S  R  D  N

                                                                PstI           NotI       XbaI
GCGAAAAACACCGTGTACCTGCAAATGAACAGTCTGAAACCGGAAGACACGGCCGTTTATTACGCTGCAGCGGTTTCCAGCGGCCGCTAATCTAGATAGGTAATCTCTGCT
A  K  N  T  V  Y  L  Q  M  N  S  L  K  P  E  D  T  A  V  Y  Y  A  A  A  V  S  S  G  R  *

                    Terminator
TAAAAGCACAGAATCTAAGATCCCTGCCATTTGGCGGGGATTTTTTTATTTGTTTTCA
```

His-tagged random sequences

```
Lac operator               NheI
AATTGTGAGCGGATAACAATTTGATGTGCTAGCGCATATCCAGTGTAGTAAGGCAAGTCCCTTCAAGAGTTATCGTTGATACCCCTCGTAGTGCACATTCCTTTAACGCTT

                                                            NdeI
CAAAATCTGTAAAGCACGCCATATCGCCGAAAGGCACACTTAATTATTAAGAGGTAATACCATATGCGGGGTTCTCATCATCATCATCATCATGGTATGGCTAGCATGACT
                                                               M  R  G  S  H  H  H  H  H  H  G  M  A  S  M  T

               BamHI
GGTGGACAGCAAATGGGATCCNNNNNNNNNNNNNNNNNNNNNNNNNNNNNNNNNNNNNNNNNNNNNNNNNNNNNNNNNNNNGGTGGAGGCGGGTCTGGGGGCGGAGGTTCA
G  G  Q  Q  M  G  S  X  X  X  X  X  X  X  X  X  X  X  X  X  X  X  X  X  X  X  X  G  G  G  G  S  G  G  G  G  S

 NotI                shot47                              XbaI                                           Terminator
GGCGGCCGCTAAGTGGTGAGGTATATTGGCGCCTTCGTGGAATGTCAGTGCCTCACCATTCTAGATAGGTAATCTCTGCTTAAAAGCACAGAATCTAAGATCCCTGCCATT
G  G  R  *

TGGCGGGGATTTTTTTATTTGTTTTCA
```

pHishot12

```
Lac operator               NheI
AATTGTGAGCGGATAACAATTTGATGTGCTAGCGCATATCCAGTGTAGTAAGGCAAGTCCCTTCAAGAGTTATCGTTGATACCCCTCGTAGTGCACATTCCTTTAACGCTT

                                                            NdeI
CAAAATCTGTAAAGCACGCCATATCGCCGAAAGGCACACTTAATTATTAAGAGGTAATACCATATGCGGGGTTCTCATCATCATCATCATCATGGTATGGCTAGCATGACT
                                                               M  R  G  S  H  H  H  H  H  H  G  M  A  S  M  T

                                                            HindIII
GGTGGACAGCAAATGGGTCGGGATCTGTACGACGATGACGATAAGGATCGATGGGGATCTAAGCTTCAGCCGGTGCTGCATCAACCGCCAGCAATGTCCTCGGCCCTGGGC
G  G  Q  Q  M  G  R  D  L  Y  D  D  D  D  K  D  R  W  G  S  K  L  Q  P  V  L  H  Q  P  P  A  M  S  S  A  L  G

ACCACGATCCGCCTGACCGCGACCCTGCGCAACGACCATGACATCGGTGTGTACAGCGTCTACTGGTACCAGCAGCGTCCGGGCCACCCTCCGCGCTTCCTGCTGCGTTAT
T  T  I  R  L  T  A  T  L  R  N  D  H  D  I  G  V  Y  S  V  Y  W  Y  Q  Q  R  P  G  H  P  P  R  F  L  L  R  Y

TTCTCGCAATCTGACAAGAGCCAGGGTCCGCAGGTCCCACCTCGCTTCTCTGGCTCCAAAGACGTCGCCCGTAACCGCGGTTATTTGAGCATCTCTGAGCTCCAACCGGAG
F  S  Q  S  D  K  S  Q  G  P  Q  V  P  P  R  F  S  G  S  K  D  V  A  R  N  R  G  Y  L  S  I  S  E  L  Q  P  E

GACGAGGCTATGTATTACGCGGCAATGGGTGCCCGTAGCTCTGAAAAAGAGGAACGTGAGCGCGAATGGGAGGAAGAAATGGAGCCGACCGCAGCCCGTACCCGTGTCCCG
D  E  A  M  Y  Y  A  A  M  G  A  R  S  S  E  K  E  E  R  E  R  E  W  E  E  E  M  E  P  T  A  A  R  T  R  V  P

GGTGGTGGAGGTTCTGGTGGTGGAGGTTCTGGTGGTGGAGGTTCCGGATCCGAAGTGCAGCTAGTTGAATCTGGGGGTGGCCTGGTGCAGCCGGGCGGTTCCCTGCGTCTC
G  G  G  G  S  G  G  G  G  S  G  G  G  G  S  G  S  E  V  Q  L  V  E  S  G  G  G  L  V  Q  P  G  G  S  L  R  L

TCCGCGGCAGCCTCTGGCTTCACCTTCAGTAGCTATGCCATGAGCTGGGTTCGTCAGGCTCCGGGCAAAGGCCTGGAATGGGTCTCTGCGATCAGCGGTTCTGGCGGTTCC
S  A  A  A  S  G  F  T  F  S  S  Y  A  M  S  W  V  R  Q  A  P  G  K  G  L  E  W  V  S  A  I  S  G  S  G  G  S

ACCTACTATGCAGACAGCGTGAAAGGCCGCTTCACCATCTCCCGGGACAACTCGAAAAACACCCTGTACCTGCAAATGAACAGTCTGAAAGCGGAAGACACGGCCGTTTAT
T  Y  Y  A  D  S  V  K  G  R  F  T  I  S  R  D  N  S  K  N  T  L  Y  L  Q  M  N  S  L  K  A  E  D  T  A  V  Y

    PstI                                                                                           NotI
TACGCTGCAGCGCGTVNKVNKVNKVNKTATVNKVNKVNKVNKTTCGACTACTGGGGTCAGGGTACTCTGGTTACCGTTTCCAGCGGTAGATAAGCGGCCGCTAAGTGGTGA
Y  A  A  A  R  X  X  X  X  Y  X  X  X  X  F  D  Y  W  G  Q  G  T  L  V  T  V  S  S  G  R  *

shot47                                  XbaI                                    Terminator
GGTATATTGGCGCCTTCGTGGAATGTCAGTGCCTCACCATTCTAGATAGGTAATCTCTGCTTAAAAGCACAGAATCTAAGATCCCTGCCATTTGGCGGGGATTTTTTTATT

TGTTTTCA
```
