# Supplementary material for: Hishot Display—A New Combinatorial Display for Obtaining Target-Recognizing Peptides
Source: PLoS One. 2013 Dec 27;8(12):e83108. doi: 10.1371/journal.pone.0083108 (PMC3873924; doi:10.1371/journal.pone.0083108)
Supplement: Figure S2 — Sequences and structure of mammalian expression vectors. The structure of the pHint expression vector is shown as a schema. Sequences of the antibody gene in VIF/pHint and VISF/pHint are shown from the signal peptide of human intelectin-1 (hITLN1) to the stop codon. The main restriction enzyme or signal peptide sequences are underlined. The X in the amino acid sequences indicates an undetermined amino acid. V, N, or K of the DNA sequences indicates mixed bases as follows: V, C/G/A; N, T/C/A/G; K, T/G. (PDF) [file pone.0083108.s002.pdf]

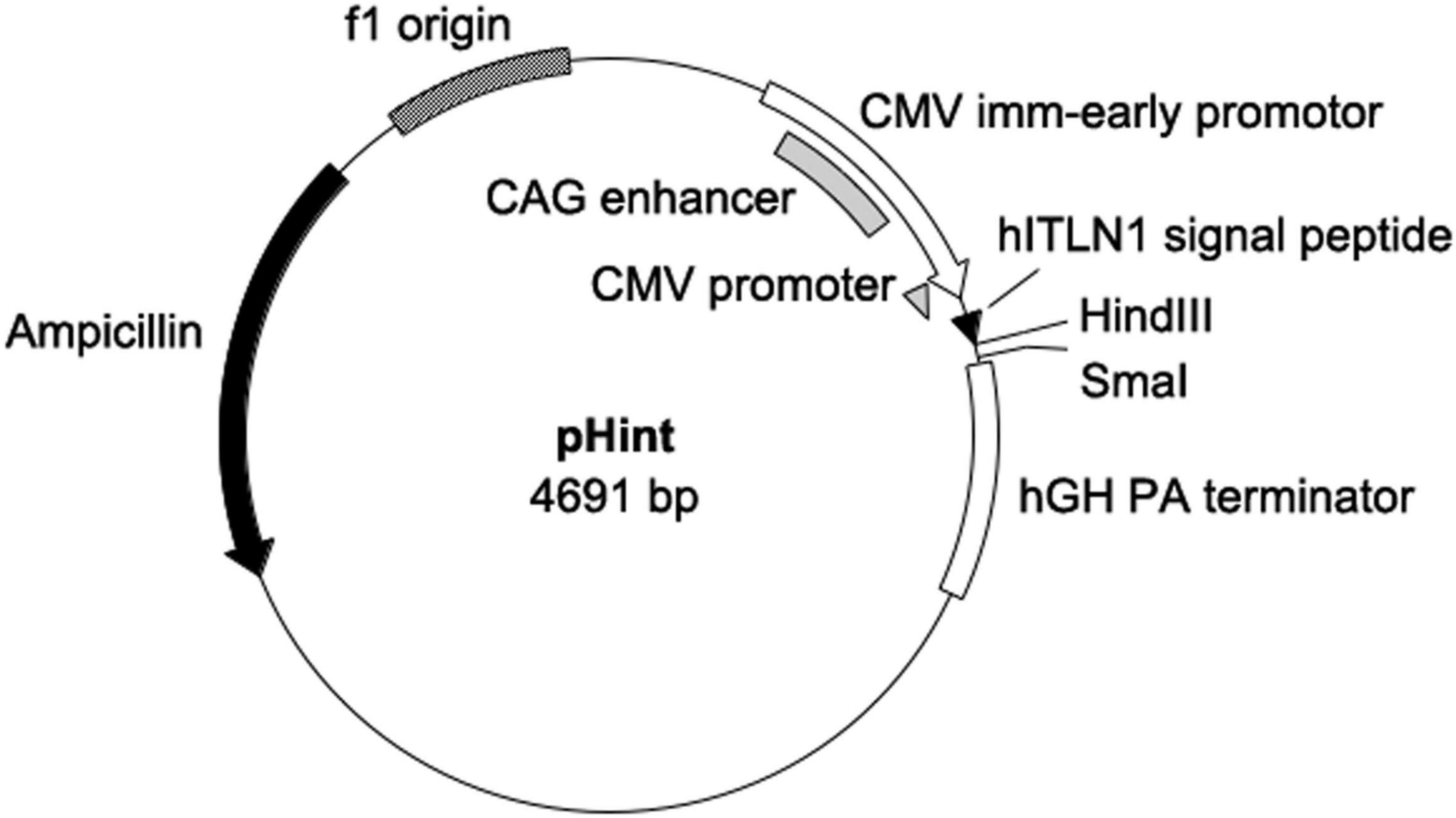
f1 origin
CMV imm-early promotor
CAG enhancer
hITLN1 signal peptide
CMV promoter
HindIII
SmaI
Ampicillin
pHint
4691 bp
hGH PA terminator

## VISF/pHint insert

```
hITLN1 signal peptide                                  HindIII
ATGAACCAACTCAGCTTCCTGCTGTTTCTCATAGCGACCACCAGAGGATGGAGTACAGATAAGCTTCAGCCGGTGCTGCATCAGCCACCTGCCATGTCCTCTGCCCTTGGA
M  N  Q  L  S  F  L  L  F  L  I  A  T  T  R  G  W  S  T  D  K  L  Q  P  V  L  H  Q  P  P  A  M  S  S  A  L  G

ACCACAATCCGCCTCACCTGCACCCTGAGGAACGACCATGACATCGGTGTGTACAGCGTCTACTGGTACCAGCAGAGGCCGGGCCACCCTCCCAGGTTCCTGCTGAGATAT
T  T  I  R  L  T  C  T  L  R  N  D  H  D  I  G  V  Y  S  V  Y  W  Y  Q  Q  R  P  G  H  P  P  R  F  L  L  R  Y

TTCTCACAATCAGACAAGAGCCAGGGCCCCCAGGTCCCCCCTCGCTTCTCTGGATCTAAAGATGTGGCCAGGAACAGGGGGTATTTGAGCATCTCTGAGCTCCAGCCTGAG
F  S  Q  S  D  K  S  Q  G  P  Q  V  P  P  R  F  S  G  S  K  D  V  A  R  N  R  G  Y  L  S  I  S  E  L  Q  P  E

GACGAGGCTATGTATTACTGTGCTATGGGGGCCCGCAGCTCCGAGAAGGAGGAAAGGGAGAGAGAATGGGAGGAAGAGATGGAACCCACCGCAGCCAGGACACGGGTCCCT
D  E  A  M  Y  Y  C  A  M  G  A  R  S  S  E  K  E  E  R  E  R  E  W  E  E  E  M  E  P  T  A  A  R  T  R  V  P

GGAGGTGGAGGCTCTGGTGGAGGAGGTAGCGGCGGTGGAGGTAGTGGATCCGAAGTGCAGCTGGTTGAGTCTGGGGGAGGCTTGGTACAGCCTGGGGGGTCCCTGAGACTC
G  G  G  G  S  G  G  G  G  S  G  G  G  G  S  G  S  E  V  Q  L  V  E  S  G  G  G  L  V  Q  P  G  G  S  L  R  L

TCCTGTGCAGCCTCTGGATTCACCTTCAGTAGCTATGCCATGAGCTGGGTCCGCCAGGCTCCAGGGAAGGGGCTGGAGTGGGTCTCAGCTATTAGTGGTAGTGGTGGTAGC
S  C  A  A  S  G  F  T  F  S  S  Y  A  M  S  W  V  R  Q  A  P  G  K  G  L  E  W  V  S  A  I  S  G  S  G  G  S

                                                                                                      PstI
ACATACTACGCAGACTCCGTGAAAGGCCGGTTCACCATCTCCAGAGACAATTCCAAAAACACGCTGTATCTGCAAATGAACAGCCTGAAAGCCGAGGACACTGCAGTTTAT
T  Y  Y  A  D  S  V  K  G  R  F  T  I  S  R  D  N  S  K  N  T  L  Y  L  Q  M  N  S  L  K  A  E  D  T  A  V  Y

                                                                                 NotI
TACTGTGCAGCGCGTVNKVNKVNKVNKTATVNKVNKVNKVNKTTCGACTACTGGGGTCAGGGTACTCTGGTTACCGTTTCCAGCGGCCGCGTTGCACCCTCGACATGCAGC
Y  C  A  A  R  X  X  X  X  Y  X  X  X  X  F  D  Y  W  G  Q  G  T  L  V  T  V  S  S  G  R  V  A  P  S  T  C  S

AAGCCCATGTGCCCACCCCCTGAACTCCTGGGGGGACCGTCTGTCTTCATCTTCCCCCCAAAACCCAAGGACACCCTCATGATCTCACGCACCCCCGAGGTCACATGCGTG
K  P  M  C  P  P  P  E  L  L  G  G  P  S  V  F  I  F  P  P  K  P  K  D  T  L  M  I  S  R  T  P  E  V  T  C  V

GTGGTGGACGTGAGCCAGGATGACCCCGAGGTGCAGTTCACATGGTACATAAACAACGAGCAGGTGCGCACCGCCCGGCCGCCGCTACGGGAGCAGCAGTTCAACAGCACG
V  V  D  V  S  Q  D  D  P  E  V  Q  F  T  W  Y  I  N  N  E  Q  V  R  T  A  R  P  P  L  R  E  Q  Q  F  N  S  T

ATCCGCGTGGTCAGCACCCTCCCCATCGCGCACCAGGACTGGTTGAGGGGCAAGGAGTTCAAGTGCAAAGTCCACAACAAGGCACTCCCGGCCCCCATCGAGAAAACCATC
I  R  V  V  S  T  L  P  I  A  H  Q  D  W  L  R  G  K  E  F  K  C  K  V  H  N  K  A  L  P  A  P  I  E  K  T  I

TCCAAAGCCAGAGGGCAGCCCCTGGAGCCGAAGGTCTACACCATGGGCCCTCCCCGGGAGGAGCTGAGCAGCAGGTCGGTCAGCCTGACCTGCATGATCAACGGCTTCTAC
S  K  A  R  G  Q  P  L  E  P  K  V  Y  T  M  G  P  P  R  E  E  L  S  S  R  S  V  S  L  T  C  M  I  N  G  F  Y

CCTTCCGACATCTCGGTGGAGTGGGAGAAGAACGGGAAGGCAGAGGACAACTACAAGACCACGCCGACCGTGCTGGACAGCGACGGCTCCTACTTCCTCTACAGCAAGCTC
P  S  D  I  S  V  E  W  E  K  N  G  K  A  E  D  N  Y  K  T  T  P  T  V  L  D  S  D  G  S  Y  F  L  Y  S  K  L

TCAGTGCCCACGAGTGAGTGGCAGCGGGGCGACGTCTTCACCTGCTCCGTGATGCACGAGGCCTTGCACAACCACTACACGCAGAAGTCCATCTCCCGCTCTCCGGGTAAA
S  V  P  T  S  E  W  Q  R  G  D  V  F  T  C  S  V  M  H  E  A  L  H  N  H  Y  T  Q  K  S  I  S  R  S  P  G  K

           EcoRV/SmaI
TGATGGGTAAATGATGGG
*
```

## VIF/pHint insert

```
hITLN1 signal peptide                                        HindIII
ATGAACCAACTCAGCTTCCTGCTGTTTCTCATAGCGACCACCAGAGGATGGAGTACAGATAAGCTTCAGCCGGTGCTGCATCAACCGCCAGCAATGTCCTCGGCCCTGGGC
M  N  Q  L  S  F  L  L  F  L  I  A  T  T  R  G  W  S  T  D  K  L  Q  P  V  L  H  Q  P  P  A  M  S  S  A  L  G

ACCACGATCCGCCTGACCGCGACCCTGCGCAACGACCATGACATCGGTGTGTACAGCGTCTACTGGTACCAGCAGCGTCCGGGCCACCCTCCGCGCTTCCTGCTGCGTTAT
T  T  I  R  L  T  A  T  L  R  N  D  H  D  I  G  V  Y  S  V  Y  W  Y  Q  Q  R  P  G  H  P  P  R  F  L  L  R  Y

TTCTCGCAATCTGACAAGAGCCAGGGTCCGCAGGTCCCACCTCGCTTCTCTGGCTCCAAAGACGTCGCCCGTAACCGCGGTTATTTGAGCATCTCTGAGCTCCAACCGGAG
F  S  Q  S  D  K  S  Q  G  P  Q  V  P  P  R  F  S  G  S  K  D  V  A  R  N  R  G  Y  L  S  I  S  E  L  Q  P  E

GACGAGGCTATGTATTACGCGGCAATGGGTGCCCGTAGCTCTGAAAAAGAGGAACGTGAGCGCGAATGGGAGGAAGAAATGGAGCCGACCGCAGCCCGTACCCGTGTCCCG
D  E  A  M  Y  Y  A  A  M  G  A  R  S  S  E  K  E  E  R  E  R  E  W  E  E  E  M  E  P  T  A  A  R  T  R  V  P

GGTGGTGGAGGTTCTGGTGGTGGAGGTTCTGGTGGTGGAGGTTCCGGATCCGAAGTGCAGCTAGTTGAATCTGGGGGTGGCCTGGTGCAGCCGGGCGGTTCCCTGCGTCTC
G  G  G  G  S  G  G  G  G  S  G  G  G  G  S  G  S  E  V  Q  L  V  E  S  G  G  G  L  V  Q  P  G  G  S  L  R  L

TCCGCGGCAGCCTCTGGCTTCACCTTCAGTAGCTATGCCATGAGCTGGGTTCGTCAGGCTCCGGGCAAAGGCCTGGAATGGGTCTCTGCGATCAGCGGTTCTGGCGGTTCC
S  A  A  A  S  G  F  T  F  S  S  Y  A  M  S  W  V  R  Q  A  P  G  K  G  L  E  W  V  S  A  I  S  G  S  G  G  S

ACCTACTATGCAGACAGCGTGAAAGGCCGCTTCACCATCTCCCGGGACAACTCGAAAAACACCCTGTACCTGCAAATGAACAGTCTGAAAGCGGAAGACACGGCCGTTTAT
T  Y  Y  A  D  S  V  K  G  R  F  T  I  S  R  D  N  S  K  N  T  L  Y  L  Q  M  N  S  L  K  A  E  D  T  A  V  Y

    PstI                                                                                         NotI
TACGCTGCAGCGCGTVNKVNKVNKVNKTATVNKVNKVNKVNKTTCGACTACTGGGGTCAGGGTACTCTGGTTACCGTTTCCAGCGGCCGCGTTGCACCCTCGACATGCAGC
Y  A  A  A  R  X  X  X  X  Y  X  X  X  X  F  D  Y  W  G  Q  G  T  L  V  T  V  S  S  G  R  V  A  P  S  T  C  S

AAGCCCATGTGCCCACCCCCTGAACTCCTGGGGGGACCGTCTGTCTTCATCTTCCCCCCAAAACCCAAGGACACCCTCATGATCTCACGCACCCCCGAGGTCACATGCGTG
K  P  M  C  P  P  P  E  L  L  G  G  P  S  V  F  I  F  P  P  K  P  K  D  T  L  M  I  S  R  T  P  E  V  T  C  V

GTGGTGGACGTGAGCCAGGATGACCCCGAGGTGCAGTTCACATGGTACATAAACAACGAGCAGGTGCGCACCGCCCGGCCGCCGCTACGGGAGCAGCAGTTCAACAGCACG
V  V  D  V  S  Q  D  D  P  E  V  Q  F  T  W  Y  I  N  N  E  Q  V  R  T  A  R  P  P  L  R  E  Q  Q  F  N  S  T

ATCCGCGTGGTCAGCACCCTCCCCATCGCGCACCAGGACTGGTTGAGGGGCAAGGAGTTCAAGTGCAAAGTCCACAACAAGGCACTCCCGGCCCCCATCGAGAAAACCATC
I  R  V  V  S  T  L  P  I  A  H  Q  D  W  L  R  G  K  E  F  K  C  K  V  H  N  K  A  L  P  A  P  I  E  K  T  I

TCCAAAGCCAGAGGGCAGCCCCTGGAGCCGAAGGTCTACACCATGGGCCCTCCCCGGGAGGAGCTGAGCAGCAGGTCGGTCAGCCTGACCTGCATGATCAACGGCTTCTAC
S  K  A  R  G  Q  P  L  E  P  K  V  Y  T  M  G  P  P  R  E  E  L  S  S  R  S  V  S  L  T  C  M  I  N  G  F  Y

CCTTCCGACATCTCGGTGGAGTGGGAGAAGAACGGGAAGGCAGAGGACAACTACAAGACCACGCCGACCGTGCTGGACAGCGACGGCTCCTACTTCCTCTACAGCAAGCTC
P  S  D  I  S  V  E  W  E  K  N  G  K  A  E  D  N  Y  K  T  T  P  T  V  L  D  S  D  G  S  Y  F  L  Y  S  K  L

TCAGTGCCCACGAGTGAGTGGCAGCGGGGCGACGTCTTCACCTGCTCCGTGATGCACGAGGCCTTGCACAACCACTACACGCAGAAGTCCATCTCCCGCTCTCCGGGTAAA
S  V  P  T  S  E  W  Q  R  G  D  V  F  T  C  S  V  M  H  E  A  L  H  N  H  Y  T  Q  K  S  I  S  R  S  P  G  K

            EcoRV/SmaI
TGATGGGTAAATGATGGG
*
```
